# Evidence for a Role of Endocannabinoids, Astrocytes and p38 Phosphorylation in the Resolution of Postoperative Pain

**DOI:** 10.1371/journal.pone.0010891

**Published:** 2010-05-28

**Authors:** Matthew S. Alkaitis, Carlos Solorzano, Russell P. Landry, Daniele Piomelli, Joyce A. DeLeo, E. Alfonso Romero-Sandoval

**Affiliations:** 1 Neuroscience Center at Dartmouth, Dartmouth Medical School, Lebanon, New Hampshire, United States of America; 2 Nuffield Department of Clinical Laboratory Sciences, University of Oxford, Oxford, United Kingdom; 3 Departments of Pharmacology and Biological Chemistry, University of California Irvine, Irvine, California, United States of America; 4 Department of Anesthesiology, Dartmouth Medical School, Lebanon, New Hampshire, United States of America; 5 Department of Pharmacology and Toxicology, Dartmouth Medical School, Lebanon, New Hampshire, United States of America; Virginia Commonwealth University, United States of America

## Abstract

**Background:**

An alarming portion of patients develop persistent or chronic pain following surgical procedures, but the mechanisms underlying the transition from acute to chronic pain states are not fully understood. In general, endocannabinoids (ECBs) inhibit nociceptive processing by stimulating cannabinoid receptors type 1 (CB_1_) and type 2 (CB_2_). We have previously shown that intrathecal administration of a CB_2_ receptor agonist reverses both surgical incision-induced behavioral hypersensitivity and associated over-expression of spinal glial markers. We therefore hypothesized that endocannabinoid signaling promotes the resolution of acute postoperative pain by modulating pro-inflammatory signaling in spinal cord glial cells.

**Methodology/Principal Findings:**

To test this hypothesis, rats receiving paw incision surgery were used as a model of acute postoperative pain that spontaneously resolves. We first characterized the concentration of ECBs and localization of CB_1_ and CB_2_ receptors in the spinal cord following paw incision. We then administered concomitant CB_1_ and CB_2_ receptor antagonists/inverse agonists (AM281 and AM630, 1 mg.kg^−1^ each, i.p.) during the acute phase of paw incision-induced mechanical allodynia and evaluated the expression of glial cell markers and phosphorylated p38 (a MAPK associated with inflammation) in the lumbar dorsal horn. Dual blockade of CB_1_ and CB_2_ receptor signaling prevented the resolution of postoperative allodynia and resulted in persistent over-expression of spinal Glial Fibrillary Acidic Protein (GFAP, an astrocytic marker) and phospho-p38 in astrocytes. We provide evidence for the functional significance of these astrocytic changes by demonstrating that intrathecal administration of propentofylline (50 µg, i.t.) attenuated both persistent behavioral hypersensitivity and over-expression of GFAP and phospho-p38 in antagonist-treated animals.

**Conclusions/Significance:**

Our results demonstrate that endocannabinoid signaling via CB_1_ and CB_2_ receptors is necessary for the resolution of paw incision-induced behavioral hypersensitivity and for the limitation of pro-inflammatory signaling in astrocytes following surgical insult. Our findings suggest that therapeutic strategies designed to enhance endocannabinoid signaling may prevent patients from developing persistent or chronic pain states following surgery.

## Introduction

Following surgical procedures such as hernia repair, breast surgery, thoracotomy, cesarean section or coronary artery bypass surgery, patients develop acute postoperative pain that is characterized by mechanical hypersensitivity (pain due to ambulation, cough or manipulation of the surgical incision area). While this acute postoperative pain typically resolves, 10-50% of patients experience persistent postsurgical pain despite analgesic treatment, and 2-10% of patients develop severe chronic pain (rates depend on the procedure) [1]. The clinical treatment of persistent or chronic pain is frequently complicated by the limited efficacy and undesirable side effects of currently available analgesic drugs. The development of safer, more effective analgesics for the management of persistent postoperative pain requires a better understanding of the mechanisms by which tissue injury-induced acute pain can develop into chronic pain.

In general, stimulation of the G protein-coupled cannabinoid receptors types 1 and 2 (CB_1_ and CB_2_) results in inhibition of nociceptive signaling pathways (reviewed in [2]). Plant-derived and synthetic CB_1_ or CB_2_ receptor agonists produce well-described antinociceptive effects [3], [4], [5], but endogenous cannabinoid compounds, or endocannabinoids (ECBs), have also gained attention for their ability to modulate pain pathways. The two main ECBs, anandamide (AEA) and 2-arachidonoylglycerol (2-AG), inhibit nociception following exogenous administration [6], [7] and have been shown to mediate stress-induced [8] and fear-conditioned [9] analgesia. Inhibitors of endocannabinoid reuptake [10], [11] or degradation [12], [13], [14] also produce antinociceptive effects. Based on these findings, it has been suggested that the endocannabinoid system mediates an adaptive response aimed at reducing pain and inflammation in response to injury or stress [15]. We therefore hypothesized endocannabinoid signaling is necessary to prevent the perpetuation of acute postoperative pain following surgical insult.

To test this hypothesis, we used a model of postoperative pain in rats that consists of a small incision made on the plantar surface of one hind paw [16]. Following paw incision, animals exhibit significant mechanical allodynia and an associated increase in the expression of glial markers, both of which spontaneously resolve over the course of approximately one week [16], [17]. Based on our previous findings that intrathecal administration of a CB_2_ receptor agonist reverses both behavioral hypersensitivity and associated over-expression of glial markers resulting from paw incision [3], we further hypothesized that endocannabinoid signaling contributes to the resolution of postoperative pain by limiting pro-inflammatory responses in spinal cord glial cells.

In the current study, we first characterized tissue concentrations of ECBs and the overall expression and cellular localization of CB_1_ and CB_2_ receptors in the spinal cord following paw incision. To test our main hypothesis, we then introduced a dual blockade of CB_1_ and CB_2_ receptors during the acute phase of paw incision-induced mechanical allodynia. Using this approach, we demonstrated that ECB signaling plays a functional role in the resolution of postoperative pain and in regulating the expression of glial cell markers and phosphorylated p38 (a MAPK associated with inflammation [18]).

## Results

### Mechanical Allodynia and Expression of Glial Markers following Paw Incision

**Figure 1:**
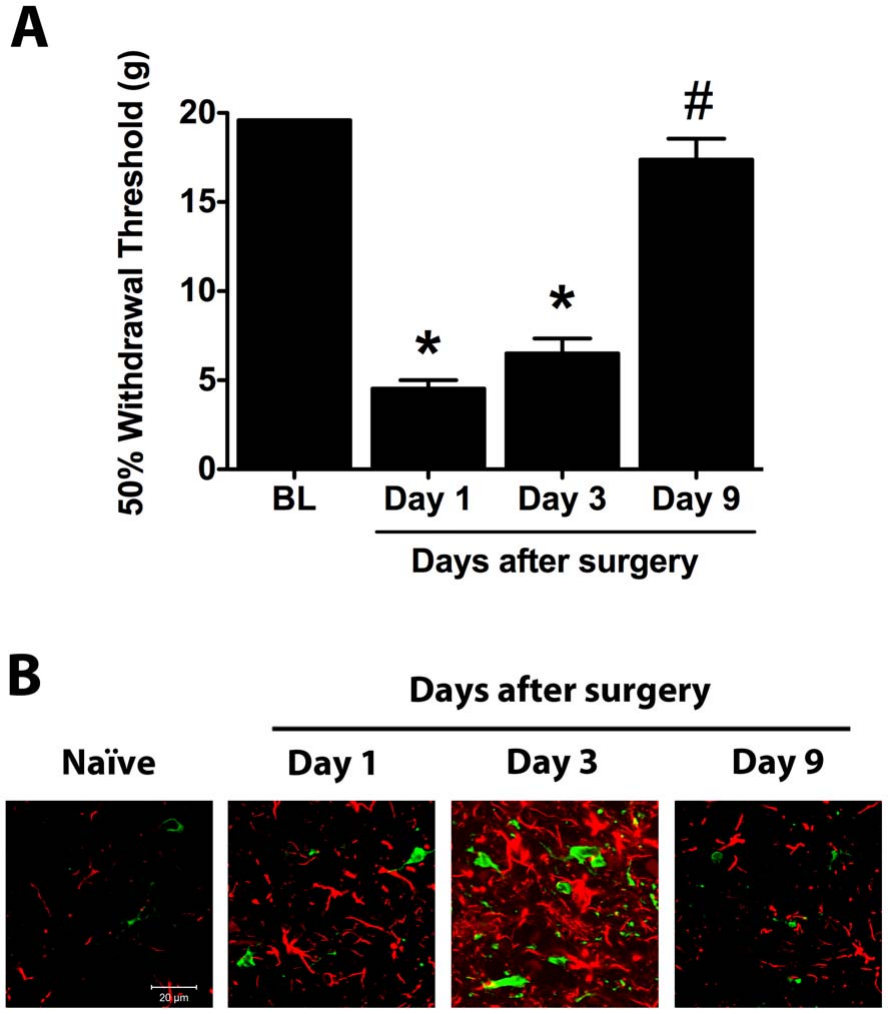
Paw incision-induced mechanical allodynia and increased glial marker expression spontaneously resolve. (A) 50% paw withdrawal thresholds were evaluated in naïve animals and ipsilateral to paw incision in animals at days 1, 3 and 9 after surgery (n = 8 per group). *p<0.05 vs. baseline values by one-way ANOVA repeated measures followed by Dunnett's post test. #p<0.05 vs. postoperative day 1 values by one-way ANOVA repeated measures followed by Dunnett's post test. (B) L5 spinal cord sections were stained for Iba-1 (green) and GFAP (red). Representative confocal images show detail of the superficial laminae of the L5 dorsal horn in naïve animals and ipsilateral to paw incision in rats at days 1, 3 and 9 after surgery. BL: Baseline.

Animals were tested for mechanical allodynia at days 1, 3 and 9 following surgery to establish a behavioral basis for the characterization of ECB levels and spinal expression of CB_1_ and CB_2_ receptor in response to paw incision. Withdrawal thresholds of the injured paw (ipsilateral to paw incision) were significantly reduced on days 1 and 3 after surgery compared to baseline values. By day 9 after paw incision, however, this mechanical hypersensitivity had resolved (Figure 1A). Similarly, no significant differences were found between baseline withdrawal thresholds and those observed on day 15 after surgery (19.04±0.4 g vs. 17.64±0.1 g, p>0.05). On all postoperative days tested, mechanical withdrawal thresholds of the uninjured paw (contralateral to paw incision) did not significantly differ from baseline values (data not shown). In agreement with our previous quantitative findings [17], both Ionized Calcium–Binding Adapter Molecule 1 (Iba-1, microglial marker) and Glial Fibrillary Acidic Protein (GFAP, astrocytic marker) demonstrated increased immunostaining in the ipsilateral dorsal horn of the L5 spinal cord on postoperative days 1 and 3 but returned to basal levels by day 9 (Figure 1B). The number of ED2/CD163-positive cells in the ipsilateral dorsal horn did not significantly change following paw incision (data not shown).

### Endocannabinoid Concentrations in the Spinal Cord and PAG following Paw Incision

**Figure 2:**
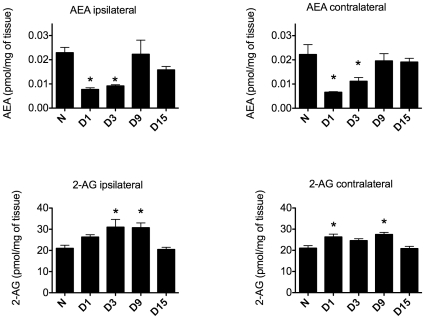
Spinal endocannabinoid levels are dynamically regulated following paw incision. Spinal cord concentrations of AEA and 2-AG were determined in the lumbar spinal cord of naïve rats (N, n = 3) and in the ipsilateral and contralateral lumbar spinal cord of rats at days 1 (D1), 3 (D3), 9 (D9) and 15 (D15) after paw incision surgery (n = 6 for each group). *p<0.05 vs. naïve group by one-way ANOVA followed by Dunnett's post test. 2-AG: 2-Arachidonoylglycerol, AEA: Anandamide.

Endocannabinoids, other fatty acid ethanolamides (FAEs) and their *N*-acylphosphatidylethanolamine species (NAPEs) precursors were detected in the lumbar spinal cord of naïve animals and animals at days 1, 3, 9 and 15 after paw incision surgery. In animals receiving paw incision surgery, the concentrations of AEA in both the ipsilateral and contralateral lumbar spinal cord were significantly lower on postoperative days 1 and 3 compared to basal concentrations in naïve animals. On postoperative days 9 and 15, however, levels of spinal AEA were no longer significantly different from those observed in naïve animals (Figure 2). Spinal concentrations of N-arachidonoylphosphatidylethanolamine (NAPE), a precursor of AEA, demonstrated no significant differences from basal concentrations at any postoperative time point tested, indicating that the observed changes in AEA concentration may have resulted from altered rates of degradation rather than synthesis (Table S1).

In contrast, the concentration of 2-AG in the ipsilateral spinal cord did not significantly differ from basal levels on day 1 after paw incision, but was significantly higher on days 3 and 9 before returning to basal levels on day 15 (Figure 2). In the contralateral spinal cord, tissue concentrations of 2-AG were significantly increased on days 1 and 9 but did not significantly differ from basal levels on days 3 and 15 after paw incision (Figure 2). In the lumbar spinal cords of naïve animals, concentrations of 2-AG were approximately 100-fold higher than those of AEA, in agreement with previous analyses of central nervous system tissues [19], [20].

In both the contralateral and ipsilateral spinal cord, tissue concentrations of the lipid amide palmitoylethanolamide (PEA), its analog, (N-oleoylethanolamine) OEA and their respective precursors, N-palmitoylphosphatidylethanolamine (NPPE) and N-oleoylphosphatidylethanolamine (NOPE), did not significantly differ from basal values at any postoperative time point tested (Figure S1 and Table S1). The concentration of PEA in the periaqueductal gray (PAG) was significantly lower in animals at days 1, 9 and 15 (but not on day 3) after surgery than in naïve animals (Figure S2). However, compared to basal concentrations, we did not observe significant changes in NAPE, NPPE, NOPE, OEA, AEA or 2-AG in the PAG at any postsurgical time point studied (Figure S2 and Table S1).

### Spinal Cord CB_1_ and CB_2_ Receptor Expression and Cellular Localization

In order to characterize the targets of the endocannabinoid system following paw incision, we evaluated the relative staining of spinal CB_1_ and CB_2_ receptors over time and examined their cellular localization. CB_1_ receptor staining in the L5 dorsal horn was significantly reduced on postoperative days 1 and 9 and spinal CB_2_ receptor staining was significantly increased on day 1 compared to staining in naïve animals (Figures S3 and S4).

**Figure 3:**
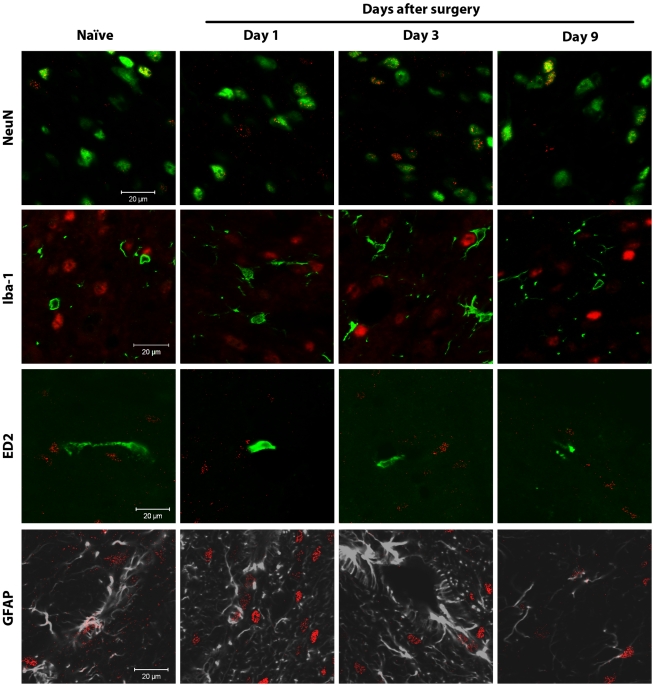
Spinal CB_1_ receptors are mainly expressed in neurons. Confocal analysis was used to determine spinal CB_1_ receptor cellular localization in the superficial laminae of the L5 dorsal horn in naïve rats or ipsilateral to surgery in rats at days 1, 3 and 9 after paw incision. Representative images are shown. CB_1_ receptor staining appears in red. NeuN (marker for neurons), Iba-1 (marker for microglia) and ED2/CD163 (ED2, marker for perivascular microglia) appear in green. GFAP (marker for astrocytes) appears in grey. In the images of CB_1_ receptors and Iba-1, CB_1_ receptor staining originally appeared green, and Iba-1 appeared in red. These colors were digitally switched in order to consistently represent CB_1_ receptor staining in red in all images. In the images of CB_1_ receptors and GFAP, GFAP staining originally appeared in green. The color of GFAP staining was digitally changed to grey in order to allow better visualization of occasional staining of CB_1_ receptors on GFAP-positive cells. The colocalization of CB_1_ receptors and NeuN staining appears in yellow.

In both naïve animals and animals receiving paw incision, spinal CB_1_ receptors were primarily expressed on NeuN-positive neurons (Figure 3). On occasion CB_1_ receptor staining also appeared to co-localize with GFAP, an astrocyte marker (Figure 3). These findings were confirmed using a 3-dimensional animation of Z-stack layered confocal images (Videos S1 and S2). Microglia (Iba-1 positive cells) and perivascular microglial cells (ED2/CD163 positive cells) did not co-localize with CB_1_ receptor staining in naïve animals or at any observed time following paw incision (Figure 3).

**Figure 4:**
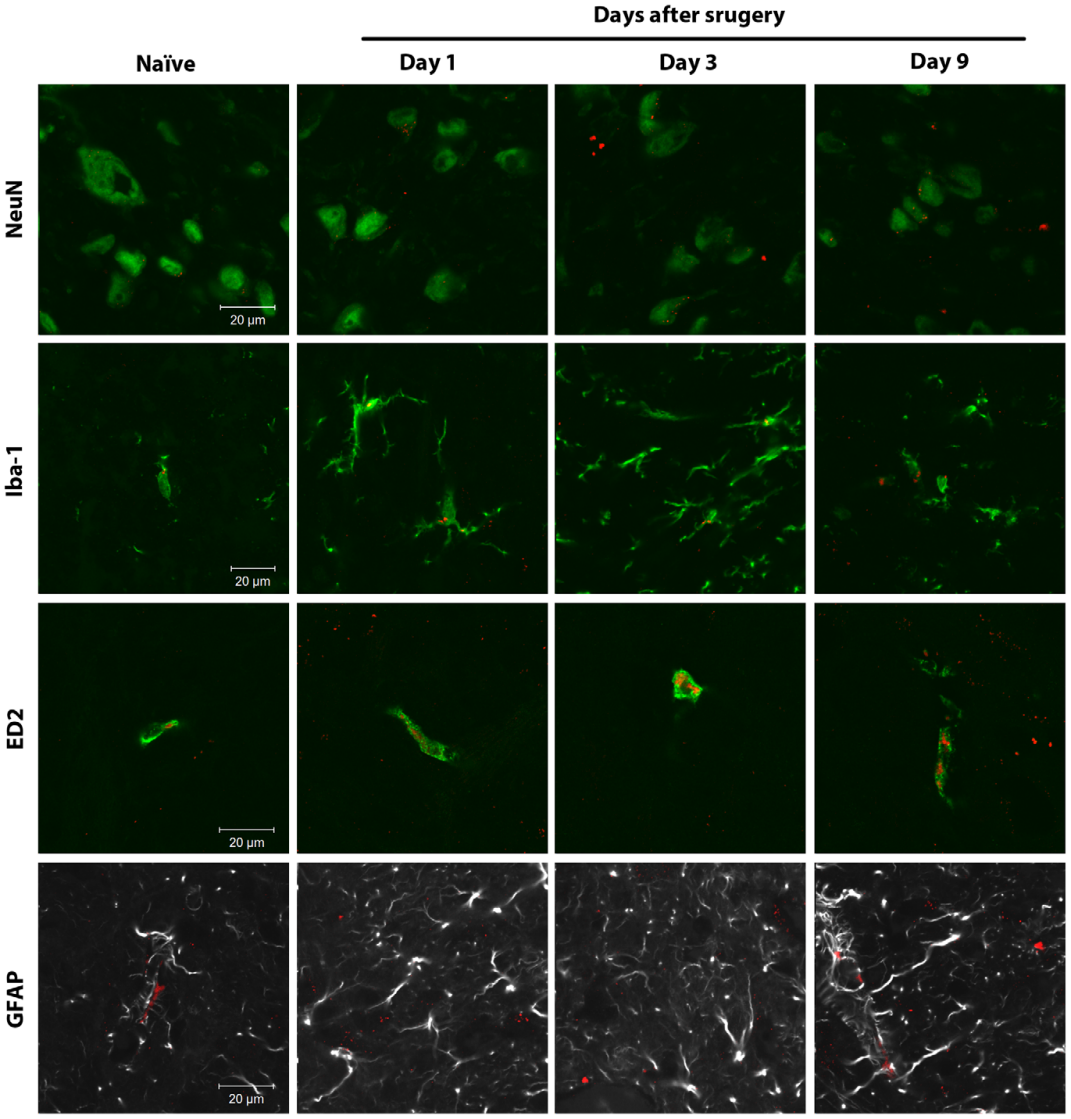
Spinal CB_2_ receptors are mainly expressed in microglial cells. Confocal analysis was used to determine CB_2_ receptor cellular localization in the superficial laminae of the L5 dorsal horn in naïve rats or ipsilateral to surgery in rats at days 1, 3 and 9 after paw incision. Representative images are shown. CB_2_ receptor staining appears in red. NeuN (marker for neurons), Iba-1 (marker for microglia) and ED2/CD163 (ED2, marker for perivascular microglia) appear in green. GFAP (marker for astrocytes) appears in grey. In the images of CB_2_ receptors and GFAP, GFAP staining originally appeared in green. The color of GFAP staining was digitally changed to grey in order to allow a better visualization of this specific marker and any potential staining of CB_2_ receptors. The colocalization of CB_2_ receptors with the other cellular markers is visualized in yellow.

Microglia (Iba-1 positive cells) and perivascular microglia (ED2/CD163 positive cells) did, however, demonstrate localized areas of strong CB_2_ receptor staining (Figure 4). CB_2_ receptor staining was occasionally observed on NeuN-positive neuronal somata but was punctate and relatively diffuse in character (Figure 4 and Video S3). GFAP-positive spinal cord astrocytes did not demonstrate CB_2_ receptor expression (Figure 4).

### Behavioral Effects of CB_1_ and CB_2_ Receptor Blockade following Paw Incision

**Figure 5:**
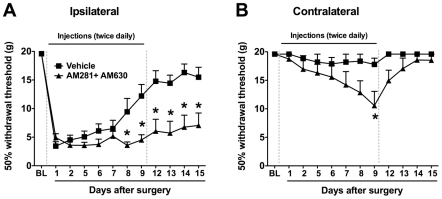
Dual blockade of CB_1_ and CB_2_ receptors prevents the normal resolution of paw incision-induced hypersensitivity. Withdrawal thresholds ipsilateral (A) and contralateral (B) to paw incision were determined before (BL: Baseline) and following surgery. Mixed antagonists of CB_1_ receptors (AM281; 1 mg.kg^−1^) and CB_2_ receptors (AM630; 1 mg.kg^−1^) or vehicle were administered twice daily (i.p.) through day 9 (doted vertical lines). *p<0.05 vs. vehicle group by two-way ANOVA repeated measures followed by Bonferroni post test. n = 8 per group.

To our knowledge, CB_1_ and CB_2_ receptors have not previously been implicated in the resolution of postoperative pain. We aimed to produce a complete blockade of ECB signaling via these receptors by systemically administering mixed CB_1_ and CB_2_ antagonists/inverse agonists (AM281 + AM630; 1 mg.kg^−1^ each, i.p.) to rats on the day of paw incision surgery and twice daily for the following nine days. Control animals were treated with vehicle according to the same paradigm. On postoperative day 1, both groups similarly demonstrated significantly lower withdrawal thresholds in the injured paw but not the uninjured paw as compared to baseline values (Figure 5). By day 8 (and subsequent days), however, the ipsilateral withdrawal thresholds of vehicle-treated animals were significantly higher than their day 1 values (p<0.05), indicating the resolution of paw incision-induced mechanical allodynia (Figure 5). In contrast, the ipsilateral withdrawal thresholds in the AM281 + AM630 group did not differ significantly from their day 1 levels at any time point tested (p>0.05) and were significantly lower than the vehicle group at day 8 and subsequent days (Figure 5). The significant difference between groups persisted through postoperative days 12-15, after administration of AM281 + AM630 had been discontinued (day 9 was the last day of treatment).

In vehicle-treated animals, the withdrawal thresholds of the uninjured paw (contralateral to paw incision) did not significantly differ from baseline values on any days tested (p>0.05). In contrast, animals treated chronically with AM281 + AM630 demonstrated a significant decrease in contralateral withdrawal thresholds on days 7, 8 and 9 compared to baseline values (p<0.05) and on day 9 compared to vehicle-treated animals (Figure 5). However, on days 12-15, after the AM281 + AM630 treatment had been discontinued, contralateral withdrawal thresholds were no longer significantly different from baseline values or from vehicle controls (Figure 5). To monitor the acute effects of AM281 + AM630 treatment, animals in preliminary trials were tested before and 30 minutes, 1 hour and 2 hours after drug or vehicle injections (n = 6 per group). On every day tested (postoperative days 1-9), AM281 + AM630 injection produced no significant changes in mechanical withdrawal thresholds at these acute time points (data not shown).

### Effect of CB_1_ and CB_2_ Receptor Blockade on spinal GFAP, Iba-1 and ED2/CD163 following Paw Incision

**Figure 6:**
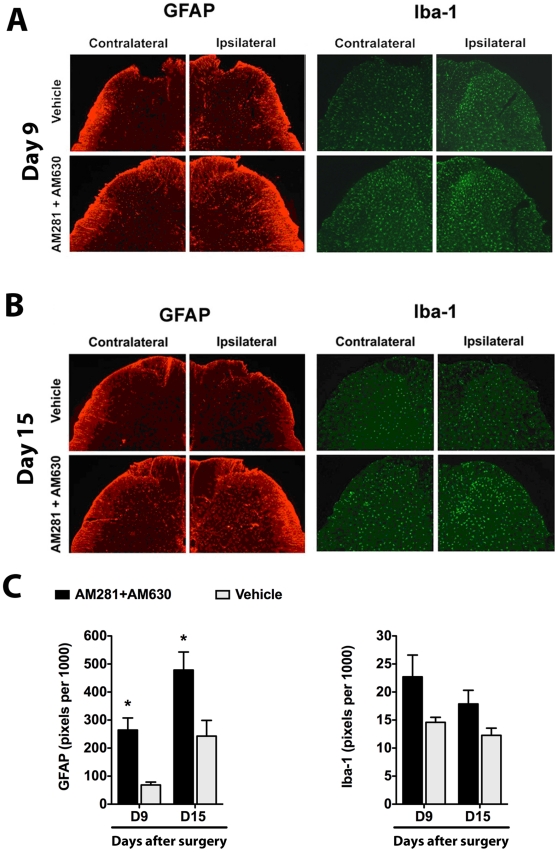
Dual blockade of CB_1_ and CB_2_ receptors results in persistent over-expression of GFAP. Representative images show GFAP (astrocytic marker) and Iba-1 (microglial marker) staining in the L5 dorsal horn ipsilateral and contralateral to paw incision in vehicle- and AM281 + AM630-treated rats at postoperative days 9 (A) and 15 (B). Quantification of these markers in laminae I-II is shown in the bottom panel (C). Staining was quantified as the number of pixels above a set threshold per total pixels in the selected area. Vehicle group: Iba-1-day 9, n = 4; Iba-1-day 15, n = 8; GFAP-day 9, n = 3; GFAP-day 15, n = 8. AM281+AM630 group: Iba-1-day 9, n = 4; Iba-1-day 15, n = 6; GFAP-day 9, n = 3; GFAP-day 15, n = 6. *p<0.05 vs. vehicle group by two-way ANOVA followed by Bonferroni post test.

On day 9 following surgery, both the superficial (I-II) and deeper laminae (III-V) of the ipsilateral L5 dorsal horn demonstrated significantly stronger GFAP staining in animals treated with AM281 + AM630 than in vehicle-treated animals (Figures 6A and 6C). On day 9, GFAP staining in the contralateral dorsal horn (laminae I-II) was also significantly stronger in animals treated with AM281 + AM630 than in vehicle-treated animals (202.5±64.1 vs. 41.6±3.7 pixels per 1000, AM281 + AM630 and vehicle groups respectively, p<0.05). At the same time point we observed no significant differences between groups in Iba-1 staining of the ipsilateral (Figures 6A and 6C) or contralateral (21.4±3.7 vs. 11.5±1.0 pixels per 1000, AM281 + AM630 and vehicle groups respectively, p>0.05) L5 dorsal horn.

On postoperative day 15 (six days after AM281 + AM630 treatment was discontinued), GFAP staining remained significantly stronger in the ipsilateral L5 dorsal horn (laminae I-II) of mixed antagonist-treated animals than in vehicle-treated animals (Figure 6B and 6C) but no significant differences between groups were observed in the contralateral dorsal horn (368.1±62.1 vs. 239.1±7.7 pixels per 1000, AM281 + AM630 and vehicle group respectively, p>0.05). At this time point, Iba-1 staining did not significantly differ between groups in either the ipsilateral (Figure 6B and 6C) or contralateral (15.8±2.0 vs. 12.1±1.1 pixels per 1000, AM281 + AM630 and vehicle groups respectively, p>0.05) L5 dorsal horn. At day 15, the number of spinal ED2/CD163-positive cells did not significantly differ between groups (data not shown).

### Effect of Propentofylline in Animals Treated with Dual CB_1_ and CB_2_ Receptor Blockade

**Figure 7:**
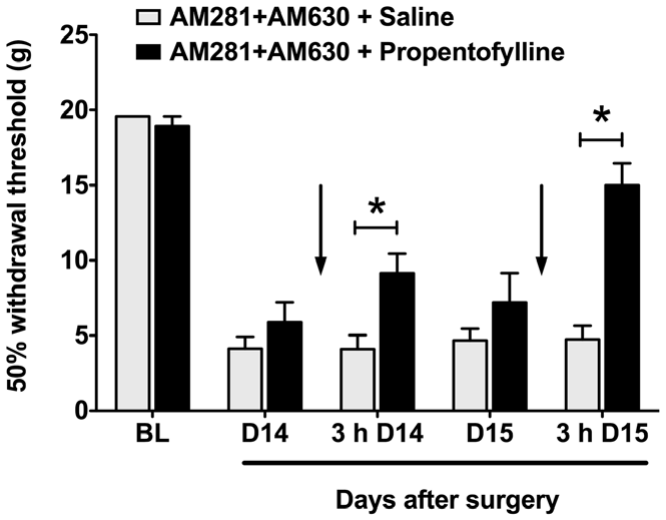
Propentofylline reverses behavioral hypersensitivity in rats treated with dual CB_1_/CB_2_ receptor blockade. 50% withdrawal thresholds were determined in AM281 + AM630-treated animals in response to subsequent propentofylline or saline treatment (indicated by arrows). Animals treated from days 1-9 with mixed CB antagonists were treated on days 14 and 15 with the glial modulator propentofylline (50 µg in 10 µl, i.t., n = 7) or saline (10 µl, i.t., n = 7). Behavior was tested before and 3 hours (3h) after injections as shown. *p<0.05 vs. saline group by two-way ANOVA repeated measures followed by Bonferroni post tests. BL: Baseline.

We then sought to evaluate whether the observed astrocytic response played a functional role in the persistent hypersensitivity observed in animals treated chronically with AM281 + AM630. We confirmed that concomitant AM281 + AM630 administered from the day of surgery to postoperative day 9 delayed the resolution of paw incision-induced allodynia through postoperative day 15. We also confirmed that after 9 days this treatment induced significant allodynia in the uninjured paw. On postoperative days 14 and 15, we then treated a subset of animals from the AM281 + AM630 group intrathecally (i.t.) with propentofylline (50 µg; hereafter referenced as the ‘AM281 + AM630/PPF group’) or vehicle (saline; hereafter referenced as the ‘AM281 + AM630/Saline group’). Propentofylline is an atypical methylxanthine that has been shown to attenuate both mechanical allodynia and associated increases in glial marker and algesic factor expression in a model of neuropathic pain [21], [22], [23]. Three hours after injection on both days that propentofylline or vehicle was administered (days 14 and 15), animals in the AM281 + AM630/PPF group displayed ipsilateral withdrawal thresholds that were significantly than AM281 + AM630/Saline controls (Figure 7). Three hours after treatment on day 15, the ipsilateral withdrawal thresholds of AM281 + AM630/PPF animals were not significantly different from pre-surgery baseline values (Figure 7).

**Figure 8:**
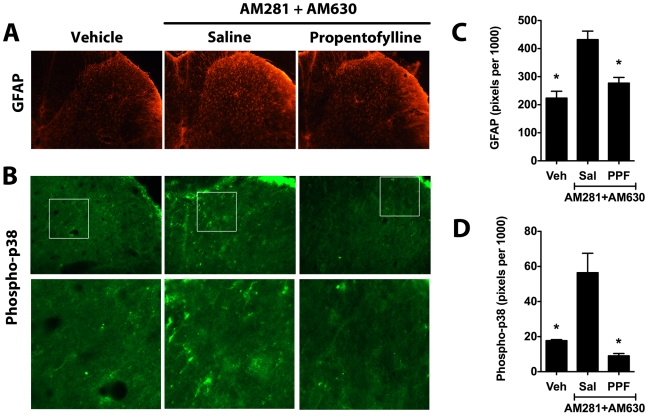
Propentofylline reverses over-expression of GFAP and phospho-p38 in rats treated with dual CB_1_/CB_2_ receptor blockade. Representative images show spinal GFAP (A) and phospho-p38 staining (B) in the ipsilateral L5 dorsal horn of animals treated from days 1-9 with AM281 + AM630 and subsequently treated on days 14 and 15 with the glial modulator propentofylline (PPF, 50 µg in 10 µl, i.t., n = 4) or saline (Sal, 10 µl, i.t., n = 3). Staining of GFAP (C) and phospho-p-38 (D) was quantified as the number of pixels above a set threshold per total pixels in the selected area. Controls (Veh, n = 3) were treated with the antagonist vehicle for nine days but did not receive either propentofylline or saline treatment. *p<0.05 vs. AM281 + AM630/saline group by one-way ANOVA followed by Dunnett's post test.

**Figure 9:**
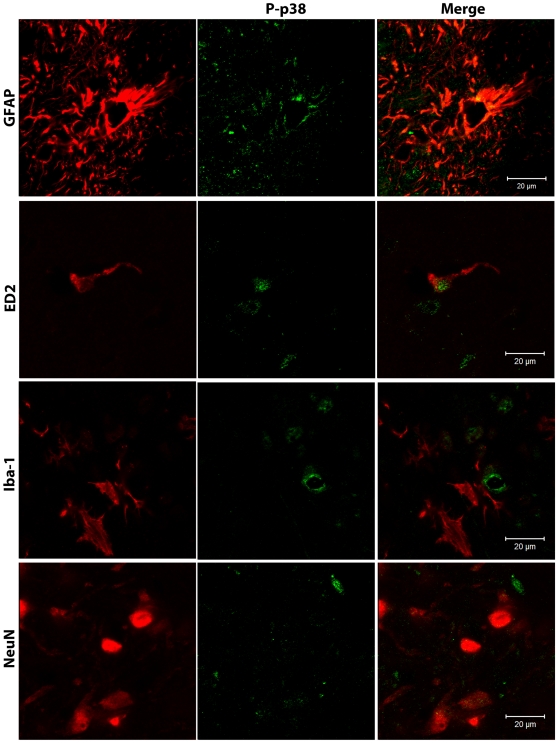
Phospho-p38 is expressed in astrocytes and perivascular microglia. Confocal analysis was used to determine phospho-p38 (P-p38) cellular localization in the superficial laminae of the ipsilateral L5 dorsal horn in AM281 + AM630-treated rats on day 15 after paw incision. Representative images are shown. Phospho-p38 appears in green. GFAP (marker for astrocytes), ED2/CD163 (ED2, marker for perivascular microglia), Iba-1 (marker for microglia) and NeuN (marker for neurons) appear in red. The color of ED2/CD163, Iba-1 and NeuN staining was digitally changed from green to red, and phospho-p38 from red to green for consistency in presenting the data and in order to allow a better visualization of the co-localization of these markers. The colors of the GFAP/phospho-p38 co-stain (top panel) were not altered.

We then used immunostaining to evaluate the effects of propentofylline treatment on relative expression of GFAP and the phosphorylated form of p38, a mitogen activated protein kinase (MAPK) associated with a pro-inflammatory cellular phenotype [18]. In addition to producing a persistent increase in spinal GFAP staining (Figure 8), treatment with AM281 + AM630 also resulted in increased phospho-p38 staining (Figure 8). Using confocal microscopy, we observed that phospho-p38 was expressed mainly in astrocytes (GFAP-positive cells) but was also expressed in perivascular microglia (ED2/CD163-positive cells, Figure 9). Spinal cord sections from the AM281 + AM630/PPF group demonstrated significantly lower GFAP and pospho-p38 staining compared to sections from AM281 + AM630/Saline animals, indicating that propentofylline treatment had reversed the persistent increase in spinal GFAP expression and p38 phosphorylation induced by the dual CB_1_/CB_2_ receptor blockade (Figure 8).

## Discussion

Despite widespread recognition of the role of endocannabinoids in regulating pain pathways, the role of the ECB system in the resolution of acute pain following peripheral tissue damage has not, to our knowledge, been previously described. In the current study we used a rat model of postoperative pain to demonstrate that dual blockade of CB_1_ and CB_2_ receptor signaling prevented the resolution of postoperative allodynia and led to a persistent increase of spinal GFAP expression and astrocytic p38 phosphorylation. We also provide evidence for the functional significance of these astrocytic changes by demonstrating that intrathecal administration of propentofylline attenuated both persistent hypersensitivity and over-expression of GFAP and phospho-p38 in animals treated with AM281 + AM630.

Anandamide and 2-AG act mainly on CB_1_ and CB_2_ receptors to modulate both normal physiology and disease pathogenesis in a host of biological systems, including nociceptive processing (reviewed in [15]). We found that AEA was reduced in the spinal cord at the time of maximum mechanical hypersensitivity following paw incision (postoperative days 1 and 3) and returned to basal levels by the time paw incision-induced allodynia had spontaneously resolved (postoperative days 9 and 15). In agreement with these results, previous studies using rodent models have shown that decreased ECB levels may mediate nociceptive sensitization arising from other disease states, such as bone cancer [24] or migraine [25]. We also observed that spinal cord levels of 2-AG were not reduced but rather increased during the phase of postoperative pain resolution (days 3 and 9 after surgery). Taken together, these results suggest that low levels of AEA in the spinal cord may contribute to paw incision-induced allodynia, and that the normalization of spinal AEA concentrations paired with elevated 2-AG may contribute to the spontaneous resolution of behavioral hypersensitivity. This conclusion is supported by our subsequent finding that chronic administration of mixed CB_1_ and CB_2_ receptor antagonists/inverse agonists resulted in persistent mechanical allodynia. While our approach cannot rule out the involvement of other systems, these findings strongly suggest that cannabinoid receptor signaling is required for the spontaneous resolution of postoperative pain. In agreement with our results, CB_1_ [26] and CB_2_ [27] receptor knock-out mice display increased behavioral sensitivity in various models of pain. By day 9, twice-daily treatment with mixed cannabinoid receptor antagonists resulted in significant hypersensitivity of the uninjured paw and associated increases in spinal GFAP staining of the superficial dorsal horn contralateral to injury, as compared to vehicle controls. These findings suggest that cannabinoid receptors may also play a role in the maintenance of basal nociceptive signaling. However, in the absence of injury, neither of these contralateral effects was apparent once treatment was discontinued.

The enhancement of glial marker expression and glial p38 phosphorylation is evident in rodent models of acute postoperative and neuropathic pain. In the former case, the incision-induced increases in glial marker expression and p38 phosphorylation spontaneously return to basal levels in association with the resolution of acute allodynia [3], [17], [28], [29]. Conversely, the inappropriate persistence of enhanced glial marker expression and p38 phosphorylation is associated with the generation and maintenance of chronic pain states [30], [31]. In the current study, the persistent postoperative allodynia induced by the dual CB_1_/CB_2_ receptor blockade was coupled with persistent over-expression of GFAP and astrocytic phospho-p38, suggesting that 1) lasting astrocytic changes may also contribute to persistent postoperative pain states and 2) under normal conditions, endocannabinoid signaling may drive postoperative pain resolution by directly or indirectly limiting pro-inflammatory signaling in astrocytes.

In a model of nerve-injury-induced chronic pain, propentofylline has been shown to inhibit hyperalgesia and associated p38 phosphorylation in glial cells [32], [33]. Additionally, propentofylline attenuates GFAP over-expression and increases expression of glutamate transporter-1 in astrocytes, which may prevent neuronal sensitization resulting from excess synaptic glutamate [23], [34]. In the current study, propentofylline attenuated both persistent hypersensitivity and over-expression of GFAP and phospho-p38 in antagonist-treated animals. These results suggest that the persistence of enhanced astrocytic GFAP and phospho-p38 expression is functionally linked to the behavioral effects of the dual cannabinoid receptor blockade. In the spinal nerve ligation model of neuropathic pain, it has been previously shown that the duration of behavioral hypersensitivity is reduced in GFAP knockout mice and that intrathecal administration of a GFAP antisense oligonucleotide reverses hypersensitivity [35]. Although these findings implicate a functional role of GFAP in neuropathic pain, it is also possible that these effects are due to mechanisms upstream (such as nitric oxide, glutamate or substance P signaling [36]) or downstream (such as myelination, blood–brain barrier integrity or astrocyte motility [37]) from GFAP expression.

The phosphorylation of glial p38 plays a well-described role in the production of pro-inflammatory factors (including cytokines, chemokines, prostaglandins and nitric oxide) that may sensitize spinal neurons and contribute to the aberrant nociceptive activity characteristic of chronic pain conditions (reviewed in [18]). In support of this role, the dephosphorylation of p38 has been shown to reverse acute postoperative allodynia [30]. Although p38 phosphorylation has thus far been described mainly in microglia [29], [30], [33], [38], [39], p38 has also been shown to participate in the astrocytic response to a dual CB_1_/CB_2_ receptor agonist *in vitro* [40]. Together, this *in vitro* study and our current findings suggest a functional relationship between astrocytic cannabinoid receptor signaling and inhibition of p38 phosphorylation. We also observed the expression of both phospho-p38 and CB_2_ receptors in perivascular cells, suggesting that ECBs may also directly regulate p38 signaling in these cells. Since the number of ED2-positive perivascular cells was not changed by the dual cannabinoid receptor blockade, our findings support the idea that an accurate evaluation of glial cell phenotype or glial reactivity cannot be obtained by exclusively studying glial marker expression. These findings also demonstrate the need for further investigation of the individual contributions of CB_1_ and CB_2_ receptor signaling to the resolution of postoperative pain. The significant effects of intrathecal propentofylline administration on mechanical allodynia and GFAP and phopho-p38 expression support the established hypothesis that spinal cord mechanisms are integral for the maintenance of persistent and chronic pain. However, based on reports of the importance of peripheral cannabinoid receptors in the induction of analgesia [26], [41], the respective roles of peripheral and central cannabinoid receptors also deserve further attention in the context of postoperative pain resolution.

Previous studies have demonstrated direct effects of cannabinoid compounds on astrocytic function [40], [42], [43]. In particular, evidence of ECB-dependent astrocytic-neuronal cross-talk [42] suggests a mechanism by which the cannabinoid receptor blockade may result in persistent pro-inflammatory signaling in astrocytes and contribute to the perpetuation of nociceptive signaling. Functional interactions between microglia and astrocytes may also play a role in mediating the astrocytic response to changes in ECB signaling. Even though astrocytes do not express CB_2_ receptors, genetic deletion of CB_2_ receptors results in enhanced GFAP expression in rodent models of neuropathic pain [27] and Huntington's disease [44]. Additionally, we have previously shown that central administration of a CB_2_ receptor agonist reduced paw incision-induced GFAP expression [3]. Taken together, these findings suggest that direct effects on astrocytes, altered neuronal-astrocytic cross-talk and/or altered microglial-astrocytic interactions may drive the persistent astrocytic response that resulted from the dual cannabinoid receptor blockade.

In conclusion, we have identified CB_1_ and CB_2_ receptor-mediated endocannabinoid signaling as a novel mechanism underlying the spontaneous resolution of acute postoperative pain. Our data further suggest that ECB signaling may contribute to the resolution of postoperative pain by limiting p38 phosphorylation and thereby inhibiting pro-inflammatory signaling in spinal astrocytes. These results provide a rationale for further investigation of the role of endocannabinoid signaling in the pathogenesis of other conditions that may be driven by aberrant glial responses, such as neurodegenerative diseases [44]. Our findings also suggest that the dysregulation of ECB signaling may contribute to the transition from acute to chronic pain states experienced by a significant portion of surgery patients. Based on these conclusions, we suggest that therapeutic strategies designed to enhance ECB signaling may reduce the incidence of persistent postoperative pain. Further studies are also warranted to evaluate whether these strategies might prove effective in preventing nerve injury from developing into chronic neuropathic pain or in treating established chronic pain with better efficacy and fewer side-effects than currently available analgesics.

## Materials and Methods

### Animals and Surgeries

Efforts were made to limit animal distress and to use the minimum number of animals necessary to achieve statistical significance. All protocols were previously approved by the Institutional Animal Care and Use Committee at Dartmouth College and in accordance with the Guidelines for Animal Experimentation of the International Association for the Study of Pain (IASP). Paw incision was used as model of postoperative pain in rats. The hypersensitivity induced in this model has been shown to largely resolve by a week after surgery [16], [17]. In this model, male Sprague-Dawley rats (Harlan, Indianapolis, IN) weighing approximately 250 g at the start of surgery underwent paw incision surgery as previously described [16]. Briefly, animals were anesthetized with 4% isoflurane in oxygen by inhalation followed by maintenance with 2% isoflurane in oxygen. Following sterilization with 10% providone-iodine solution, a 1 cm midline incision was made on the plantar face of the left hind paw from the heel to the base of the toes using a No. 10 scalpel blade and sterile technique. A small forceps was used to elevate the flexor tendon from the heel to the toes and to irritate it for 6-8 seconds. The wound was cleaned and two inverted 6-0 silk sutures were used to close the incision. Animals were housed individually and maintained in a 12:12 h light/dark cycle with *ad libitum* access to food and water. Separate sets of animals received paw incision surgery for the ECB tissue concentration studies, the CB_1_ and CB_2_ receptor expression and cellular localization studies and the cannabinoid receptor blockade experiments.

### Endocannabinoid Tissue Concentration

Untreated animals received paw incision surgery and were subjected to deep inhalation of 4% isoflurane in oxygen and euthanized by decapitation on days 1, 3, 9 and 15 after surgery (n = 6 each). Naïve animals (n = 3) were used as the control group. The lumbar enlargement of the spinal cord was collected and dissected with a scalpel to divide the ipsilateral and contralateral sides (left and right for naïve animals). The periaqueductal gray (PAG) was also harvested. Tissues were wrapped in foil and snap frozen on dry ice and removed to -80 °C until analysis. Frozen tissues were weighed and homogenized in methanol (1 mL/100 mg of tissue) containing [^2^H_4_]FAEs, [^2^H_8_] 2-AG (Cayman Chemical, Ann Arbor, MI) and 1-O-Hexadecyl-2-palmitoyl-sn-glycero-3-phospho-(N-palmitoyl)-ethanolamide (Enzo Life Sciences International, Inc., Plymouth Meeting, PA) as internal standards. [^2^H_4_]FAEs were synthesized as previously described [45]. Lipids were extracted with chloroform and water and were fractionated by open-bed silica gel column chromatography as previously described [46]. FAEs and 2-AG were quantified as previously described [47] using an 1100-LC system coupled to a 1946A-MS detector (Agilent Technologies, Inc., Palo Alto, CA) equipped with an electrospray ionization interface. Quantifications were conducted using an isotope dilution method, monitoring the sodium adduct of the molecular ions ([M+Na]^+^). We quantified NAPEs by LC-MS^n^ using an 1100-LC system (Agilent Technologies) equipped with an Ion Trap XCT (Agilent Technologies) as previously described [47]. Tissue-derived NAPEs were identified by comparison of their LC retention times and MS^n^ fragmentation patterns with those of authentic standards, prepared as previously described [47]. Extracted ion chromatograms were used to quantify each NAPE precursor ion by monitoring the characteristic lyso-NAPE product ions in MS^2^ using 1-O-Hexadecyl-2-palmitoyl-sn-glycero-3-phospho-(N-palmitoyl)-ethanolamide (*m/z* 914.8>676.8) as an internal standard. The following NAPEs were monitored: 1-stearoyl, 2-arachidonoyl-*sn*-glycero-3-phosphoethanolamine-*N*-palmitoyl (m/z 1004.8>718.8), 1-stearoyl, 2-arachidonoyl-*sn*-glycero-3-phosphoethanolamine-*N*-oleoyl (m/z 1030.8>744.6), and 1-stearoyl, 2- docosahexaenoyl-*sn*-glycero-3-phosphoethanolamine-*N*-arachidonoyl (m/z 1076.8>766.8). Detection and analysis were controlled by Agilent/Bruker Daltonics software version 5.2.

### Drug Treatment

Animals in the experimental group were treated with intraperitoneal (i.p.) injections of AM281 (1-(2,4-dichlorophenyl)-5-(4-iodophenyl)-4-methyl-*N*-4-morpholinyl-1*H*-pyrazole-3-carboxamide; Tocris, Ellisville, Missouri) and AM630 (6-iodo-2-methyl-1-[2-(4-morpholinyl)ethyl]-1*H*-indol-3-yl](4-methoxyphenyl)methanone; Tocris, Ellisville, Missouri) at a dose of 1 mg.kg^−1^ each in 500 µl [41], [48], [49]. AM281 is a diarylpyrazole analog CB_1_ receptor antagonist/inverse agonist [50], [51]. AM630 is an aminoalkylindole analogue CB_2_ receptor antagonist/inverse agonist [52], [53], [54]. Although some studies indicate that CB_1_ receptor antagonists may interact with TRPV1 channels [55] or with the opioid system via prodynorphin production [49], it is unlikely that our behavioral findings were influenced by interactions between our antagonist treatment and these systems, which do not play a role in paw-incision-induced hypersensitivity [56], [57]. AM281 and AM630 were concomitantly administered in the morning between 10:00 – 11:00 AM and in the evening between 4:00 – 5:00 PM on the day of surgery and the following nine days. A control group was injected with an equivalent volume of vehicle (12% DMSO in 500 µl saline). Animals that were perfused on day 9 did not receive drug or vehicle injections that day. A subset of animals received i.t. injections of the glial modulator propentofylline (50 µg in 10 µl) or saline (10 µl) on days 14 and 15 after surgery.

### Behavioral Testing

To test mechanical allodynia, calibrated von Frey filaments (Stoelting, Wood Dale, IL) were pressed against the plantar aspect of each hind paw for approximately 6 seconds. The 50% withdrawal thresholds of each hind paw were determined twice at 10-15 minute intervals using the up-down statistical method [58]. The average of these values was used for data analysis. The untreated animals that provided tissue for the ECB concentration and CB_1_ and CB_2_ receptor expression and cellular localization studies were tested for baseline values, received paw incision surgery and were tested before tissue collection on postoperative days 1, 3, 9 or 15. In both cases, the naïve animals used for controls were also tested for baseline behavior but did not receive paw incision surgery. In the cannabinoid receptor blockade experiments, animals receiving mixed antagonist or vehicle treatment (see above) were tested twice before surgery to obtain a baseline then daily (before the morning injection) over the course of the 15 days following surgery to monitor the resolution of hypersensitivity. In a subset of animals, behavior was tested before antagonist administration and at 30 minutes, 1 hour and 2 hours after injection; however, since drug administration produced no acute effects (data not shown), daily behavioral tests for the majority of animals were only performed once daily (directly before A.M. drug injections). In the subset of animals that were additionally injected intrathecally (i.t.) with either propentofylline (n = 7) or saline (n = 7) as described above, behavior was tested before and 3 hours after i.t. injections. During all behavioral testing, the experimenter was blinded to experimental conditions.

### Tissue Preparation for Immunofluorescence and Immunohistochemistry

In order to examine expression of Iba-1 (a microglial marker), GFAP (an astrocytic marker), ED2/CD163 (a perivascular cell marker) and CB_1_/CB_2_ receptor and CB_1_/CB_2_ receptor co-localization with other cell markers in response to paw incision, untreated animals received paw incision surgery and were perfused as described below on days 1, 3 and 9 after surgery (n = 4 each). Naïve animals (n = 4) were used as the control group. In separate experiments, animals receiving mixed CB_1_ and CB_2_ receptor antagonists (day 9 after surgery, n = 4; day 15 after surgery, n = 6) or vehicle (day 9 after surgery, n = 4; day 15 after surgery, n = 8) were perfused as described below on days 9 and 15 post-surgery. These animals were used to provide spinal cord tissue for the analysis of ED2/CD163, Iba-1 and GFAP expression for the cannabinoid receptor blockade experiments. Another set of animals received vehicle (n = 3), mixed CB_1_ and CB_2_ receptor antagonists followed by propentofylline on days 14 and 15 (n = 4) or mixed CB_1_ and CB_2_ receptor antagonists followed by saline vehicle on days 14 and 15 (n = 4). Animals were perfused 3 hours after propentofylline or saline treatment as described below on day 15 post-surgery. These animals were used to provide spinal cord tissue for the analysis of GFAP expression and p38 phosphorylation.

In all cases, animals were first subjected to deep inhalation of 4% isoflurane in oxygen and perfused transcardially with 0.01 M phosphate buffered saline (PBS; approximately 150 ml) followed by 4% formaldehyde (350 ml or until stiff) at room temperature. After fixation, a laminectomy was performed, the lumbar enlargement was removed and the L5 segment was separated and cryoprotected in 30% sucrose for 48–72 h at 4°C. The tissue was then mounted and frozen in Optimal Cutting Temperature (O.C.T.) compound (Sakura Finetek, Torrance, CA) at -80°C. Immunofluorescence was performed on transverse 20 µm sections of the L5 spinal cord. Sections to be stained for ED2/CD163 were then post-fixed with 4% formaldehyde for 5 minutes and washed 3 times with 1X PBS wash buffer (.0067 M) for 10 minutes at 4°C before blocking. This step was omitted for all other stains. All sections were blocked in 5% Normal Goat Serum (NGS) and 0.01% Triton-X-100 for 1 hour at 4°C. Sections were incubated in the appropriate primary antibody or antibodies diluted in a buffer composed of 1% NGS and 1% Triton-X-100 in PBS overnight at 4°C. Microglia were stained with a rabbit polyclonal antibody directed against Iba-1 (1∶1000, Wako Pure Chemical Industries, Richmond, VA). Astrocytes were stained with a rabbit polyclonal antibody directed against GFAP (1∶10,000, Dako Cytomation, Glostrup, Denmark) except when co-staining with Iba-1. To avoid cross-reactivity in this latter case, a mouse polyclonal antibody was used to stain GFAP (1∶400, Sigma, Saint Louis, Missouri). Perivascular cells were stained with a mouse polyclonal antibody directed against ED2/CD163 (1∶150, Serotec, Raleigh, NC). Neurons were stained with a mouse polyclonal antibody directed against Neuronal Nuclei, NeuN (1∶10,000, Chemicon, Billerica, Massachusetts). A rabbit polyclonal antibody was used to label CB_1_ receptors (1∶200, Cayman, Ann Arbor, MI; except when using the TSA Signal Amplification Kit, see below). A goat polyclonal antibody was used to label CB_2_ receptors (1∶150, Santa Cruz, Santa Cruz, CA). A mouse or rabbit monoclonal antibody was used to label phospho-p38 (1∶100, Cell Signaling, Danvers, MA). The following day, tissue sections were washed 3 times with PBS for 10 minutes at 4°C and stained with the appropriate secondary fluorescent antibody or antibodies diluted 1∶250 in a buffer composed of 1% NGS and 1% Triton-X-100 in PBS for 1 hour at 4°C. For CB_2_ receptor expression at different time points following paw incision surgery we used the Vector ELITE ABC (Vector Labs, Burlingame, CA), avidin-biotin complex technique. The following secondary antibodies were used as indicated in Table S2 (Supplemental Material): Alexa-Fluor™ 488 Goat anti-Rabbit IgG1 (Molecular Probes, Eugene, Oregon), Alexa-Fluor™ 488 Goat anti-Mouse IgG1 (Molecular Probes, Eugene, Oregon), Alexa-Fluor™ 555 Goat anti-Mouse IgG (Molecular Probes, Eugene, Oregon) and Alexa-Fluor™ 555 Donkey anti-Goat IgG (Molecular Probes, Eugene, Oregon). Alexa-Fluor™ 488 fluorophore-tagged secondary antibodies emit green wavelengths of visible light and Alexa-Fluor™ 555 fluorophore-tagged secondary antibodies emit red wavelengths of visible light. To avoid cross-reactivity between the secondary antibodies in the CB_2_ receptor co-localization experiments, sections were first incubated in Alexa-Fluor™ 555 Donkey anti-Goat IgG (Molecular Probes, Eugene, Oregon) as described above, washed 2 times in PBS and then incubated in the appropriate Alexa-Fluor™ 488 secondary antibody as described above. This protocol modification prevented binding of the Alexa-Fluor™ 555 Donkey anti-Goat IgG to the goat-derived Alexa-Fluor™ 488. To avoid cross-reactivity when co-staining with primary antibodies against Iba-1 and CB_1_ receptors that are both rabbit-derived, a TSA Signal Amplification Kit was used following the manufacturer instructions (PerkinElmer LifeSciences Inc, Boston, MA). On the first day, normal immunofluorescence protocol was followed except that sections were incubated only in anti-CB_1_ receptor antibody at a concentration of 1∶10,000. On the second day sections were washed 2 times for 5 minutes in PBS then incubated in a biotinylated Goat α Rabbit secondary antibody for 1 hour at 4°C. Sections were then subjected to another wash, incubated in SA-HRP (1∶100) for 1 hour at 4°C, washed again and incubated in the TSA fluorophore (1∶250) for 10 minutes at 4°C. Sections were then washed again and incubated overnight in the Iba-1 primary antibody (1∶1000). The next day sections were subjected to normal day 2 immunofluorescence protocol to visualize Iba-1 (described above). One control was included with only the anti-CB_1_ receptor primary antibody (1∶10,000) and the Alexa 555 Goat α Rabbit secondary antibody to control for any cross-reactivity that might cause CB_1_ receptor staining to appear in red. A second control included only the anti-CB_1_ receptor primary antibody and the TSA kit in order to visualize the staining achieved in the absence of the co-stain. Finally, a third control included the TSA kit, Iba-1 primary and the Alexa 555 Goat α Rabbit secondary antibody but excluded the anti-CB_1_ receptor primary antibody. This third control provided visualization of the non-specific background staining produced by the kit alone. All controls confirmed the specificity of the complete co-stain.

In all cases, tissue sections were washed again as before, mounted on glass slides, dehydrated, treated with Vectashield (Vector Labs, Burlingame, CA) and sealed with a coverslip and nail polish. The specificity of each antibody was tested by omitting the primary antibody on 1-3 additional sections.

### Imaging and Image Analysis

The induction of glial reactivity has been described as an increase in the number (proliferation and/or migration) and complexity of these populations (rounded cell bodies and thicker processes), resulting in an increase in the expression of glial markers. However, the role of common glial markers in determining glial phenotype is not well understood. In order to better assess the induction of inflammatory activity in CNS cell populations, we also studied the expression of the pro-inflammatory mitogen activated protein kinase (MAPK) p38 in its active form (phosphorylated) in glial and neuronal cells. In order to quantify these markers, stained sections were examined with an Olympus fluorescence microscope, and images were captured with a Q-Fire cooled camera (Olympus, Melville, NY). In the superficial laminae (I-II) and deep laminae (III-V) of the L5 dorsal horn, the intensity of CB_1_ receptor, CB_2_ receptor, Iba-1, GFAP and phospho-p38 staining was quantified as the number of pixels per area above a preset intensity threshold using SigmaScan Pro 5 (SPSS, Chicago, IL) as previously described [3], [17]. For each area, the number of high-intensity pixels was normalized to the total pixels selected in that area. For each animal, 4 sections were analyzed, and an average of these values was used for statistical analysis. To ensure the consistency of staining technique between groups, immunofluorescent/immunohistochemical staining and image capture were both performed simultaneously on all tissue samples that were directly compared and subjected to statistical tests. Due to the relatively small volume of ED2/CD163 positive cells, this marker was quantified by counting positively stained cells on an Olympus fluorescence microscope.

### Statistical Analysis

For behavioral data and comparison against base line or day 1 after surgery, one-way ANOVA repeated measures was conducted and followed by Dunnett's or Bonferroni multiple comparison post test (only when p<0.05 was found). Two-way ANOVA repeated measures was used for comparisons between groups at every time point for behavioral data, and followed by Bonferroni post tests when p<0.05 was found. To compare relative CB_1_ receptor, CB_2_ receptor and ED2/CD163 staining and tissue concentrations of ECBs, their precursors and other non-cannabinoid ethanolamides over time, one-way ANOVA followed by Dunnett's multiple comparison post test, when p<0.05 was found, was performed. Two-way ANOVA plus Bonferroni post tests when p<0.05 was used for comparisons between groups for Iba-1 or GFAP expression. All data are presented as the mean ± SEM. In all cases a p<0.05 was considered significant. GraphPad Prism 5 software was used to perform all statistical analyses.

## Supporting Information

Figure S1 Spinal OEA and PEA concentrations do not change after paw incision. Concentrations of OEA and PEA were measured in naïve rats (N, n = 3) and ipsilateral and contralateral to paw incision in rats at days 1 (D1), 3 (D3), 9 (D9) and 15 (D15) after surgery (n = 6 for each group). OEA: N-oleoylethanolamine, PEA: palmitoylethanolamide. No significant difference was found between groups using one-way ANOVA (p>0.05).
(4.47 MB TIF)

Figure S2 PEA levels in the PAG are reduced after paw incision. PAG concentrations of OEA, PEA, AEA and 2-AG were measured in naïve rats (N, n = 3) and at days 1 (D1), 3 (D3), 9 (D9) and 15 (D15) after surgery in rats receiving paw incision (n = 6 for each group). *p<0.05 vs. naive group by one-way ANOVA followed by Dunnett's post test. 2-AG: 2-Arachidonoylglycerol, AEA: Anandamide, OEA: N-oleoylethanolamine, PEA: palmitoylethanolamide.
(3.59 MB TIF)

Figure S3 CB_1_ receptor expression is reduced on days 1 and 9 after paw incision. Representative images (A) show CB_1_ receptor staining in the L5 dorsal horn of naïve rats (N, n = 3) and ipsilateral to paw incision in rats at days 1 (D1, n = 3), 3 (D3, n = 3) and 9 (D9, n = 4) after surgery. The middle panel (B) shows detail of the superficial laminae (I-II) of the dorsal horn of these spinal cord sections. Staining was quantified (C) as the number of pixels above a set threshold per total pixels in the selected area. *p<0.05 vs. naive by one-way ANOVA followed by Dunnett's post test.
(2.66 MB TIF)

Figure S4 CB_2_ receptor expression is increased on day 1 following paw incision. Representative images (A) show CB_2_ receptor staining in the L5 dorsal horn of naïve rats (N, n = 3) and ipsilateral to paw incision in rats at days 1 (D1, n = 3), 3 (D3, n = 3) and 9 (D9, n = 4) after surgery. The middle panel (B) shows detail of the superficial laminae (I-II) of the dorsal horn of these spinal cord sections. Staining was quantified (C) as the number of pixels above a set threshold per total pixels in the selected area. These representative images have been digitally transformed to black and white color. *p<0.05 vs. naive group by one-way ANOVA followed by Dunnett's post test.
(2.86 MB TIF)

Table S1 Tissue Concentrations of Fatty Acid Ethanolamide Precursors. Levels of N-acylphosphatidylethanolamine species precursors in the spinal cord and perieaqueductal grey (PAG) of naïve rats and of rats at days 1, 3, 9 and 15 after paw incision surgery. No significant differences were found between groups for any of these compounds using one-way ANOVA (p>0.05). I: ipsilateral to paw incision, C: contralateral to paw incision, NAPE: N-arachidonoylphosphatidylethanolamine, NPPE: N-palmitoylphosphatidylethanolamine, NOPE: N-oleoylphosphatidylethanolamine. Data presented as mean (s.e.m.).
(0.07 MB DOC)

Table S2 Details of antibody selections for immunohistochemistry and immunofluorescence experiments. CB_1_: Cannabinoid Type 1, CB_2_: Cannabinoid Type 2, ED2: Perivascular cell marker, GFAP: Glial Fibrillary Acidic Protein, Iba-1: Ionized Calcium-Binding Adapter Molecule 1, NeuN: Neuronal Nuclei.
(0.09 MB DOC)

Video S1 Low levels of CB_1_ receptor are expressed in spinal astrocytes. Analysis of Z-stacked confocal images was used to confirm CB_1_ receptor colocalization with GFAP-positive astrocytes in the superficial laminae of the L5 dorsal horn of naïve rats. CB_1_ receptor staining appears in red. GFAP (marker for astrocytes) appears in grey. In these images, GFAP staining originally appeared in green. The the color of GFAP staining was digitally changed to grey in order to allow better visualization of occasional staining of CB_1_ receptors on GFAP-positive cells.
(7.08 MB AVI)

Video S2 Low levels of CB_1_ receptor are expressed in spinal astrocytes at day 9 after paw incision. Analysis of Z-stacked confocal images was used to confirm CB_1_ receptor colocalization with GFAP-positive astrocytes in the superficial laminae of the L5 dorsal horn at day 9 after paw incision surgery. CB_1_ receptor staining appears in red. GFAP (marker for astrocytes) appears in grey. In these images, GFAP staining originally appeared in green. The color of GFAP staining was digitally changed to grey in order to allow better visualization of occasional staining of CB_1_ receptors on GFAP-positive cells.
(7.08 MB AVI)

Video S3 Low levels of CB_2_ receptor are expressed in neuronal somata. Analysis of Z-stacked confocal images was used to confirm CB_2_ receptor colocalization with NeuN-positive neuronal somata in the superficial laminae of the L5 dorsal horn at day 3 after paw incision surgery. CB_2_ receptor staining appears in red. NeuN (neuronal marker) appears in green.
(11.80 MB AVI)
