# Supplementary figures and images for: Evidence for a Role of Endocannabinoids, Astrocytes and p38 Phosphorylation in the Resolution of Postoperative Pain
Source: PLoS One. 2010 May 28;5(5):e10891. doi: 10.1371/journal.pone.0010891 (PMC2878341; doi:10.1371/journal.pone.0010891)

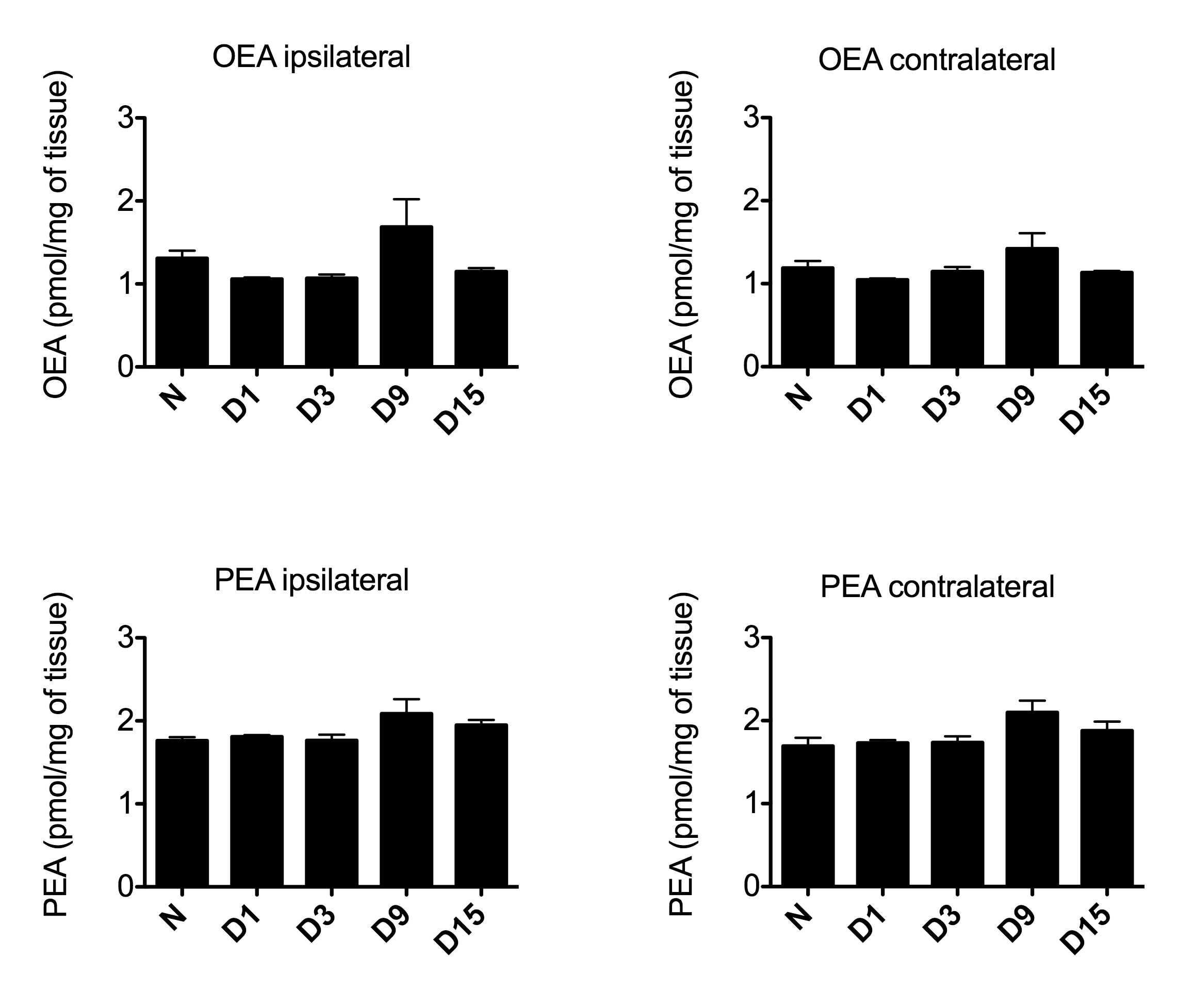

Supplement: Figure S1 — Spinal OEA and PEA concentrations do not change after paw incision. Concentrations of OEA and PEA were measured in naïve rats (N, n = 3) and ipsilateral and contralateral to paw incision in rats at days 1 (D1), 3 (D3), 9 (D9) and 15 (D15) after surgery (n = 6 for each group). OEA: N-oleoylethanolamine, PEA: palmitoylethanolamide. No significant difference was found between groups using one-way ANOVA (p>0.05). (4.47 MB TIF) [file pone.0010891.s001.tif]

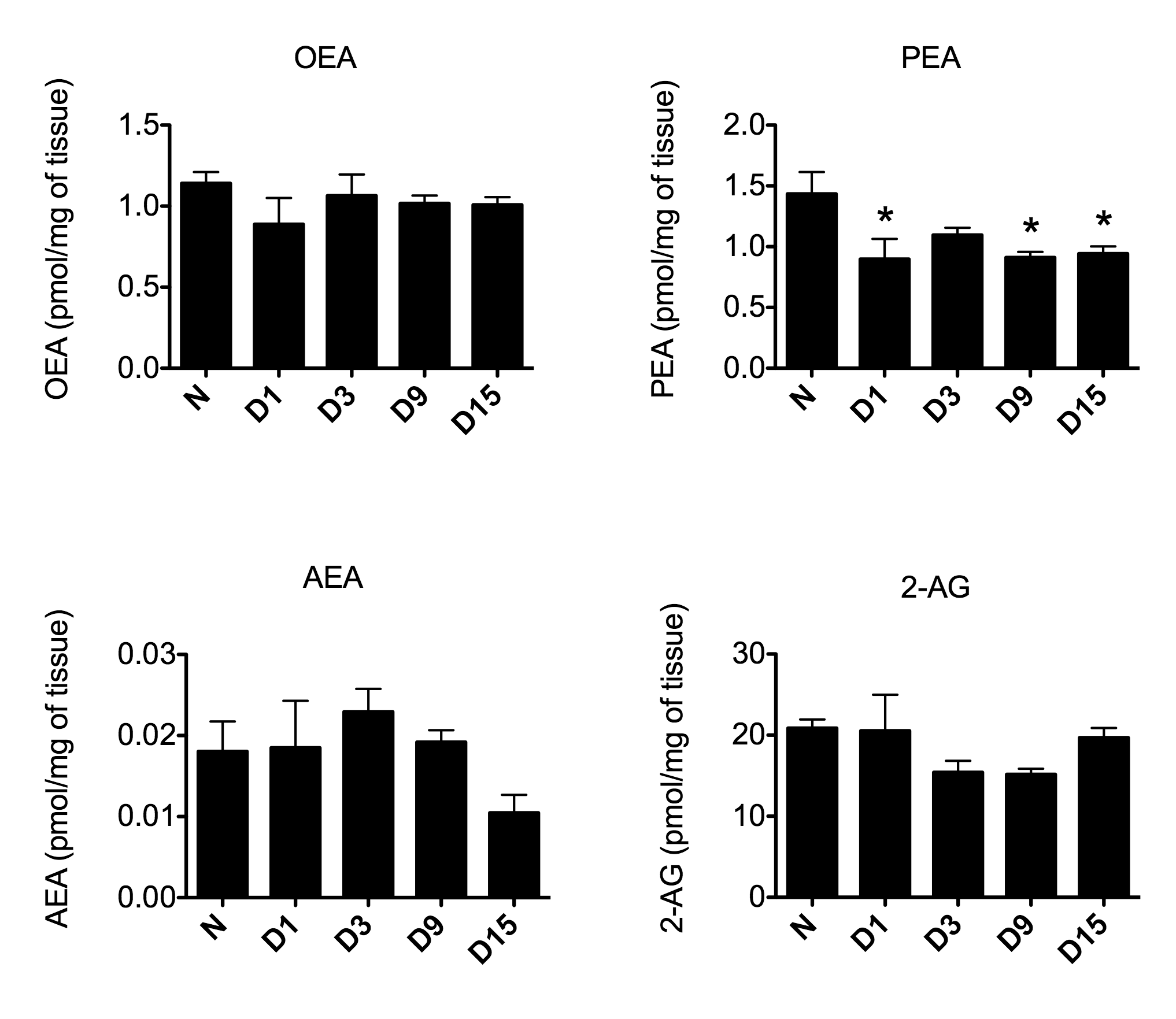

Supplement: Figure S2 — PEA levels in the PAG are reduced after paw incision. PAG concentrations of OEA, PEA, AEA and 2-AG were measured in naïve rats (N, n = 3) and at days 1 (D1), 3 (D3), 9 (D9) and 15 (D15) after surgery in rats receiving paw incision (n = 6 for each group). *p<0.05 vs. naive group by one-way ANOVA followed by Dunnett's post test. 2-AG: 2-Arachidonoylglycerol, AEA: Anandamide, OEA: N-oleoylethanolamine, PEA: palmitoylethanolamide. (3.59 MB TIF) [file pone.0010891.s002.tif]

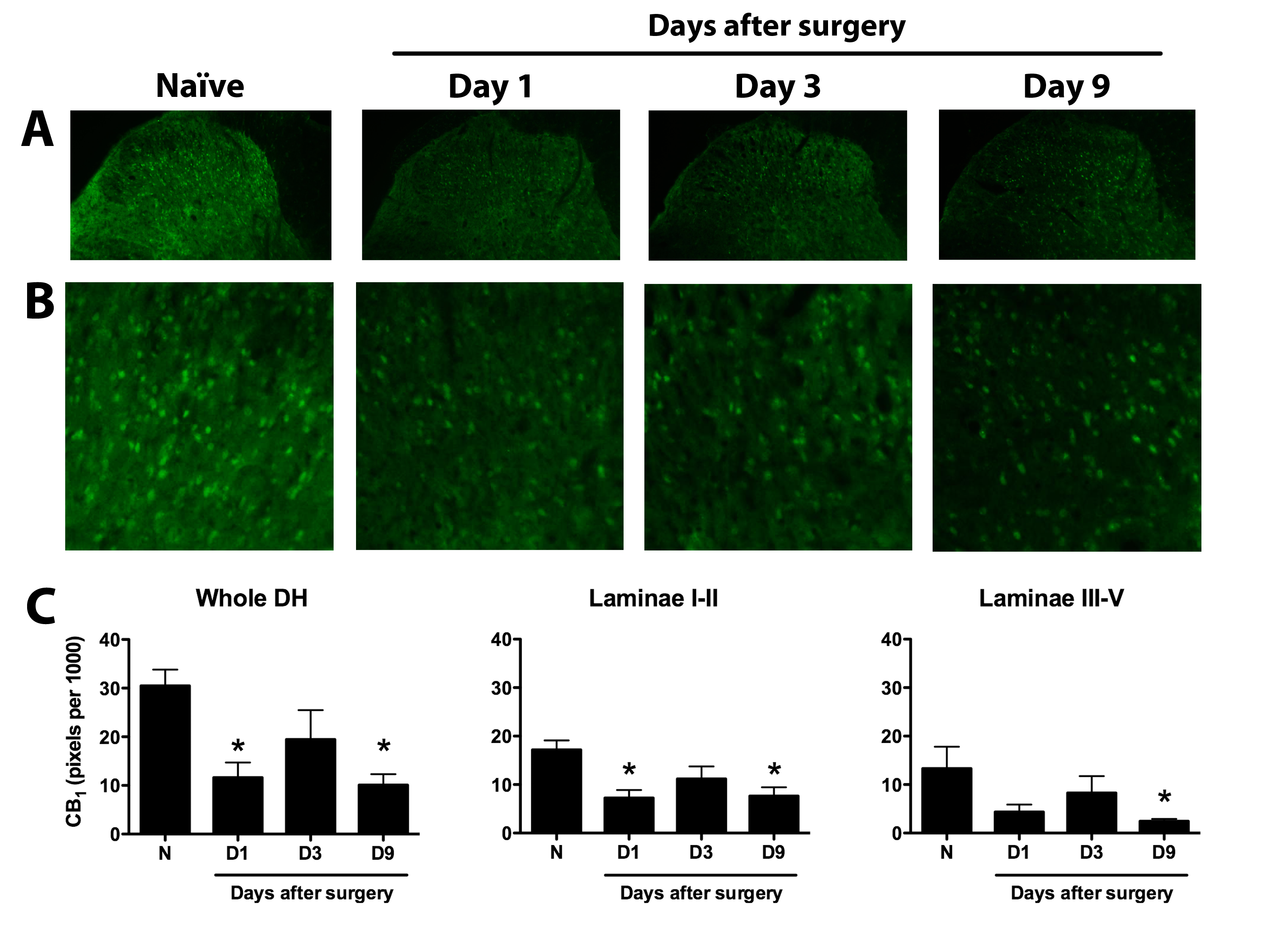

Supplement: Figure S3 — CB1 receptor expression is reduced on days 1 and 9 after paw incision. Representative images (A) show CB1 receptor staining in the L5 dorsal horn of naïve rats (N, n = 3) and ipsilateral to paw incision in rats at days 1 (D1, n = 3), 3 (D3, n = 3) and 9 (D9, n = 4) after surgery. The middle panel (B) shows detail of the superficial laminae (I-II) of the dorsal horn of these spinal cord sections. Staining was quantified (C) as the number of pixels above a set threshold per total pixels in the selected area. *p<0.05 vs. naive by one-way ANOVA followed by Dunnett's post test. (2.66 MB TIF) [file pone.0010891.s003.tif]

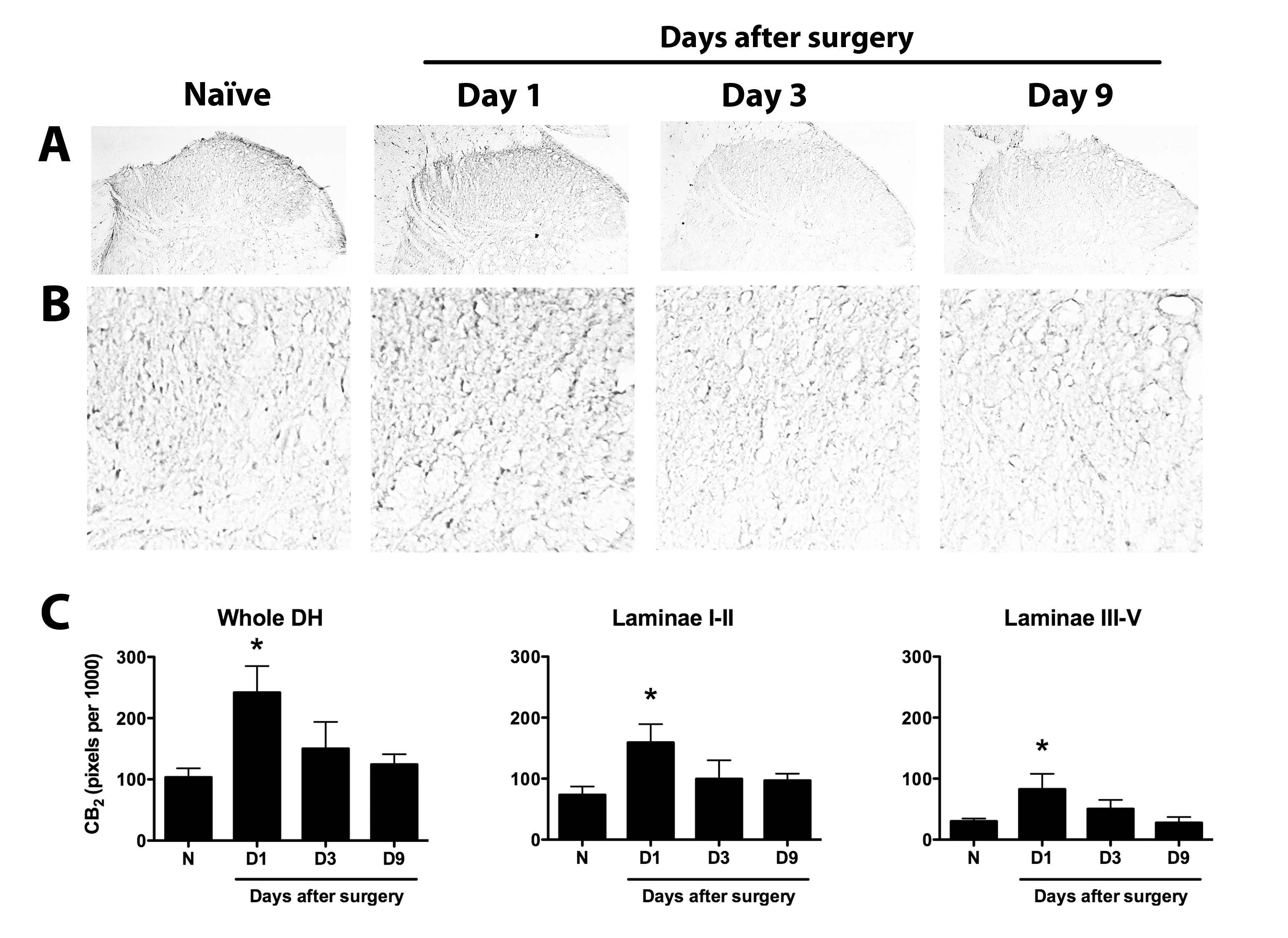

Supplement: Figure S4 — CB2 receptor expression is increased on day 1 following paw incision. Representative images (A) show CB2 receptor staining in the L5 dorsal horn of naïve rats (N, n = 3) and ipsilateral to paw incision in rats at days 1 (D1, n = 3), 3 (D3, n = 3) and 9 (D9, n = 4) after surgery. The middle panel (B) shows detail of the superficial laminae (I-II) of the dorsal horn of these spinal cord sections. Staining was quantified (C) as the number of pixels above a set threshold per total pixels in the selected area. These representative images have been digitally transformed to black and white color. *p<0.05 vs. naive group by one-way ANOVA followed by Dunnett's post test. (2.86 MB TIF) [file pone.0010891.s004.tif]
