# Supplementary material for: Evidence for a Role of Endocannabinoids, Astrocytes and p38 Phosphorylation in the Resolution of Postoperative Pain
Source: PLoS One. 2010 May 28;5(5):e10891. doi: 10.1371/journal.pone.0010891 (PMC2878341; doi:10.1371/journal.pone.0010891)
Supplement: Table S1 — Tissue Concentrations of Fatty Acid Ethanolamide Precursors. Levels of N-acylphosphatidylethanolamine species precursors in the spinal cord and perieaqueductal grey (PAG) of naïve rats and of rats at days 1, 3, 9 and 15 after paw incision surgery. No significant differences were found between groups for any of these compounds using one-way ANOVA (p>0.05). I: ipsilateral to paw incision, C: contralateral to paw incision, NAPE: N-arachidonoylphosphatidylethanolamine, NPPE: N-palmitoylphosphatidylethanolamine, NOPE: N-oleoylphosphatidylethanolamine. Data presented as mean (s.e.m.). (0.07 MB DOC) [file pone.0010891.s005.doc]

| **NAPE** | | | | | |
| --- | --- | --- | --- | --- | --- |
|  | **Naïve** | **Day 1** | **Day 3** | **Day 9** | **Day 15** |
| **Spinal Cord (I)** | 0.13 (0.06) | 0.17 (0.05) | 0.2 (0.01) | 0.27 (0.09) | 0.3 (0.04) |
| **Spinal Cord (C)** | 0.09 (0.05) | 0.16 (0.05) | 0.09 (0.03) | 0.21 (0.09) | 0.26 (0.08) |
| **PAG** | 0.08 (0.04) | 0.14 (0.11) | 0.04 (0.01) | 0.07 (0.02) | 0.02 (0.01) |

| **NPPE** | | | | | |
| --- | --- | --- | --- | --- | --- |
|  | **Naïve** | **Day 1** | **Day 3** | **Day 9** | **Day 15** |
| **Spinal Cord (I)** | 0.38 (0.12) | 0.75 (0.26) | 0.68 (0.16) | 0.78 (0.12) | 0.91 (0.18) |
| **Spinal Cord (C)** | 0.42 (0.05) | 0.44 (0.11) | 0.64 (0.16) | 0.64 (0.11) | 1.05 (0.29) |
| **PAG** | 0.26 (0.05) | 0.29 (0.04) | 0.36 (0.11) | 0.28 (0.02) | 0.31 (0.05) |

| **NOPE** | | | | | |
| --- | --- | --- | --- | --- | --- |
|  | **Naïve** | **Day 1** | **Day 3** | **Day 9** | **Day 15** |
| **Spinal Cord (I)** | 0.26 (0.07) | 0.47 (0.1) | 0.46 (0.12) | 0.51 (0.1) | 0.64 (0.17) |
| **Spinal Cord (C)** | 0.39 (0.21) | 0.41 (0.07) | 0.34 (0.07) | 0.53 (0.07) | 0.45 (0.15) |
| **PAG** | 0.18 (0.01) | 0.19 (0.04) | 0.15 (0.03) | 0.16 (0.04) | 0.17 (0.03) |
