# Supplementary material for: Evidence for a Role of Endocannabinoids, Astrocytes and p38 Phosphorylation in the Resolution of Postoperative Pain
Source: PLoS One. 2010 May 28;5(5):e10891. doi: 10.1371/journal.pone.0010891 (PMC2878341; doi:10.1371/journal.pone.0010891)
Supplement: Table S2 — Details of antibody selections for immunohistochemistry and immunofluorescence experiments. CB1: Cannabinoid Type 1, CB2: Cannabinoid Type 2, ED2: Perivascular cell marker, GFAP: Glial Fibrillary Acidic Protein, Iba-1: Ionized Calcium-Binding Adapter Molecule 1, NeuN: Neuronal Nuclei. (0.09 MB DOC) [file pone.0010891.s006.doc]

| **Antigen (Co-stain)** | **Primary antibody origin** | **Secondary antibody origin** | **Fluorophore optimal excitation (nm)** |
| --- | --- | --- | --- |
| CB_1_ | Mouse | Goat α Mouse | 488 |
| CB_2_ | Goat | Donkey α Goat | Avidin-biotin complex technique |
| ED2 | Mouse | Goat α Mouse | 488 |
| Iba1 (GFAP) | Rabbit (Mouse) | Goat α Rabbit (Goat α Mouse) | 488 (555) |
| Iba1 (CB_2_) | Rabbit (Goat) | Goat α Rabbit (Donkey α Goat) | 488 (555) |
| GFAP (CB_2_) | Rabbit (Goat) | Goat α Rabbit (Donkey α Goat) | 488 (555) |
| ED2 (CB_2_) | Mouse (Goat) | Goat α Mouse (Donkey α Goat) | 488 (555) |
| NeuN (CB_2_) | Mouse (Goat) | Goat α Mouse (Donkey α Goat) | 488 (555) |
| Iba1 (CB_1_) | Rabbit (Rabbit) | Goat α Rabbit (TSA Signal Amplification Kit) | 555 (488) |
| GFAP (CB_1_) | Mouse (Rabbit) | Goat α Mouse (Goat α Rabbit) | 488 (555) |
| ED2 (CB_1_) | Mouse (Rabbit) | Goat α Mouse (Goat α Rabbit) | 488 (555) |
| NeuN (CB_1_) | Mouse (Rabbit) | Goat α Mouse (Goat α Rabbit) | 488 (555) |
| Iba-1 (P-p38) | Rabbit (Mouse) | Goat α Rabbit (Goat α Mouse) | 488 (555) |
| ED2 (P-p38) | Mouse (Rabbit) | Goat α Mouse (Goat α Rabbit) | 488 (555) |
| GFAP (P-p38) | Mouse (Rabbit) | Goat α Mouse (Goat α Rabbit) | 555 (488) |
| NeuN (P-p38) | Mouse (Rabbit) | Goat α Mouse (Goat α Rabbit) | 488 (555) |
